# Fecal Calprotectin as a Marker of the Gut Immune System Activation Is Elevated in Parkinson’s Disease

**DOI:** 10.3389/fnins.2019.00992

**Published:** 2019-09-27

**Authors:** Agata Mulak, Magdalena Koszewicz, Magdalena Panek-Jeziorna, Ewa Koziorowska-Gawron, Sławomir Budrewicz

**Affiliations:** ^1^Department of Gastroenterology and Hepatology, Wrocław Medical University, Wrocław, Poland; ^2^Department of Neurology, Wrocław Medical University, Wrocław, Poland

**Keywords:** Parkinson’s disease, brain-gut axis, intestinal inflammation, fecal calprotectin, inflammatory marker

## Abstract

**Introduction:**

Alpha-synucleinopathy constituting a characteristic feature of Parkinson’s disease (PD) occurs at all levels of the brain-gut axis including the enteric nervous system (ENS). Lesions in the ENS may be connected with gut inflammation, increased intestinal permeability and dysmotility contributing to the pathogenesis of PD and its gastrointestinal manifestations.

**Aims:**

To evaluate fecal calprotectin and zonulin as biomarkers of gut inflammation and intestinal barrier dysfunction in PD patients.

**Methods:**

Quantitative evaluation of fecal biomarkers was performed by ELISA tests in 35 PD patients and 20 healthy controls. Additionally, patients filled out a short questionnaire concerning gastrointestinal symptoms.

**Results:**

Median fecal calprotectin level (μg/g) was significantly higher in PD patients compared to the controls: 54.5 (29.0–137.9) vs. 9.7 (5.2–23.3), *p* < 0.0001. Applying age-related reference ranges, the increased fecal calprotectin level was found in 43% of PD patients and in none of the control subjects (*p* < 0.001). No correlation between fecal calprotectin level and PD duration was observed. No statistically significant difference between the groups regarding zonulin level was found. The most frequent bowel symptoms reported by PD patients included constipation (69% of subjects), feeling of incomplete evacuation (51%), bloating (51%), abdominal pain (20%), and alternating bowel movement pattern (17%).

**Conclusion:**

The evaluation of fecal calprotectin level may be a useful tool to detect the signs of gut immune system activation present in a remarkable number of PD patients, also in the early stage of the disease. Calprotectin may constitute a critical link between amyloid formation and neuroinflammatory cascades serving as a prospective diagnostic and therapeutic target.

## Introduction

Recently, disturbances within the brain-gut-microbiota axis have been increasingly recognized in the pathophysiology of neurodegenerative disorders such as Parkinson’s disease (PD) (Mulak and Bonaz, 2015; Felice et al., 2016). Accumulating data confirm that both qualitative and quantitative alterations in the gut microbiota composition may precede or occur during the course of PD (Hasegawa et al., 2015; Keshavarzian et al., 2015; Scheperjans et al., 2015; Sampson et al., 2016; Unger et al., 2016; Bedarf et al., 2017; Hill-Burns et al., 2017; Hopfner et al., 2017; Petrov et al., 2017). There is a close relationship between gut microbiota alterations and gut inflammation associated with increased intestinal permeability (Houser and Tansey, 2017). The gut microbiota upregulates local and systemic inflammation through different mechanisms including release of lipopolysaccharides from pathogenic bacteria (Villarán et al., 2010). The peripheral immune response characterized by the presence of proinflammatory cytokines such as TNF-α, IL-1β and IL-8 in the serum may induce a disruption of the blood-brain barrier and promote microglia-mediated inflammation and neurotoxicity (Alvarez-Arellano and Maldonado-Bernal, 2014; Bodea et al., 2014). Therefore, an excessive stimulation of the innate immune system may result in systemic and central nervous system inflammation, while the initiation of alpha-synuclein (α-syn) misfolding may be related to activation of enteric neurons and enteric glial cells (Mulak and Bonaz, 2015; Houser and Tansey, 2017; Stolzenberg et al., 2017). Additionally, the adaptive immune system may be disturbed by bacterial proteins cross-reacting with human antigens and cross-seeded misfolding via the molecular mimicry pathway (Friedland, 2015). The gut bacteria are also able to produce numerous neurotransmitters and neuromodulators such as short-chain fatty acids, however, their role in neuroinflammation and neurodegeneration has not been yet fully elucidated (Mulak, 2018).

Gastrointestinal manifestations in PD, among which constipation is the most common one, frequently precede motor symptoms indicating early involvement of the digestive tract in the pathological process and supporting the concept that the gut may represent a rout of entry for a putative environmental factor (Braak et al., 2003; Felice et al., 2016). In fact, intestinal inflammation associated with increased expression of proinflammatory cytokines has been found in colonic biopsies from PD patients (Devos et al., 2013). Recently, the analysis of stool immune profiles has provided further evidence on gut inflammation in PD (Houser et al., 2018). It has been also shown that immune activation induces gut barrier dysfunction, while increased intestinal permeability correlates with levels of α-syn, deposit of which is a hallmark of PD (Forsyth et al., 2011; Kelly et al., 2014). Interestingly, there is a growing body of epidemiological, experimental and clinical data supporting the close link between PD and inflammatory bowel diseases (IBD) (Becker et al., 2019; Rolli-Derkinderen et al., 2019).

The main aim of this study was to evaluate whether fecal calprotectin applied as a useful tool in diagnosing and monitoring IBD can also detect signs of the gut immune system activation in patients with PD. Additionally, fecal zonulin level as a marker of intestinal barrier dysfunction was assessed.

## Materials and Methods

### Subjects

Thirty-five patients with PD hospitalized or consulted at the Department of Neurology at Wrocław Medical University (Poland) and 20 healthy controls were recruited in the study. The protocol of this study was approved by the local Ethics Committee (KB-491/2017). A written informed consent in accordance with the Declaration of Helsinki was obtained from all participants prior to the study enrollment.

All subjects provided stool samples. The demographic, clinical and therapeutic characteristics of PD patients are presented in Table 1. The prevalence of gastrointestinal symptoms, concomitant disorders and medications, including proton pump inhibitors (PPIs), in PD patients were assessed based on a questionnaire. The following features were considered as the exclusion criteria: inflammatory bowel disease and symptomatic diverticulosis, previous gastrointestinal surgery except for appendectomy and cholecystectomy, and use of nonsteroidal anti-inflammatory drugs or antibiotics within the last month prior the stool collection.

**TABLE 1:** Demographic, clinical and therapeutic characteristics of the recruited PD patients.

	**PD patients**	**Controls**
Number of subjects	35	20
Mean age (years) [range]	63 [38–82]	63 [52–86]
Sex (males/females)	19/16	9/11
Body Mass Index (kg/m^2^)	26.05	26.38
Mean disease duration (years) [range]	9 [1–20]	NA
Hoehn and Yahr Scale	HY stage II, *n* = 17	NA
	HY stage III, *n* = 18	
MDS UPDRS – part III M [25Q–75Q]	32 [22.3–46.3]	NA
ADL scale M [25Q–75Q]	80 [70–80]	NA
LED (mg) M [25Q–75Q]	625 [381–985]	NA

*ADL, the Schwab and England Activities of Daily Living Scale; M, median; MDS UPDRS, Movement Disorder Society Unified Parkinson’s Disease Rating Scale; NA, not applicable, LED, Levodopa Equivalent Dose.*

### Collection of Stool Samples and Quantitative Evaluation of Fecal Biomarkers

From each subject a stool sample of approximately 5 g was collected according to the instruction. All samples were delivered to the laboratory within 24 h. After required preparation the samples were stored at −20°C until processing as described previously (Ohlsson et al., 2017). The quantitative evaluations of calprotectin and zonulin in stool samples were performed by ELISA tests: EK-CAL (BÜHLMANN Laboratories, Switzerland) and IDK^®^Zonulin (Immundiagnostik AG, Germany), respectively.

### Statistical Analysis

The results are expressed as median along with the lower and upper quartiles [25Q–75Q]. Non-parametric statistics (the Mann-Whitney *U* test) were applied to compare differences in fecal calprotectin and zonulin levels between the groups. For comparison of differences in frequency of abnormal results between the groups the chi-squared test was used. Relation between the parameters was assessed using correlation analysis and the Spearman’s rank correlation coefficient (R) was calculated.

## Results

**FIGURE 1:**
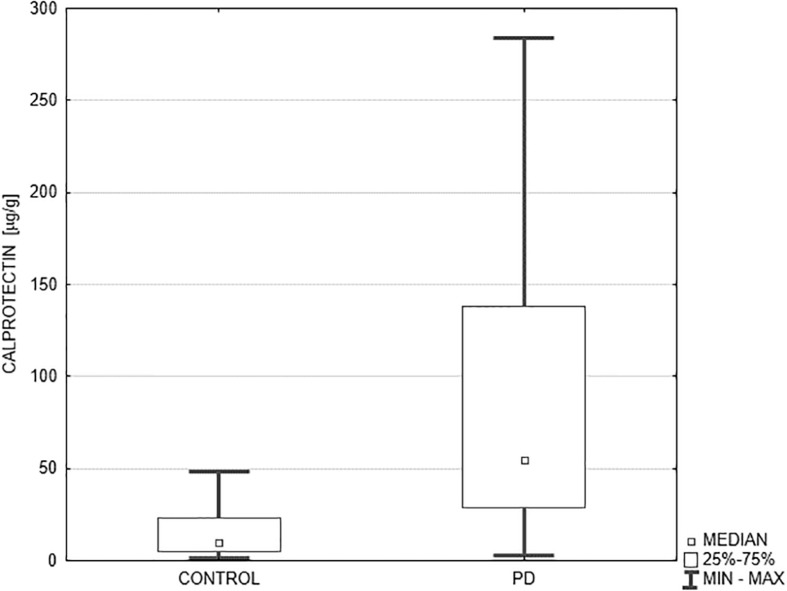
Comparison of fecal calprotectin level in PD patients and the controls. Median fecal calprotectin level (μg/g) expressed as median and the lower and upper quartiles was significantly higher in PD patients (*n* = 35) compared to the controls (*n* = 25): 54.5 (29.0–137.9) vs. 9.7 (5.2–23.3), *p* < 0.0001. PD, Parkinson’s disease.

The median fecal calprotectin level (μg/g) was significantly higher in PD patients compared to the controls: 54.5 (29.0–137.9) vs. 9.7 (5.2–23.3), *p* < 0.0001 (Figure 1). Additionally, we evaluated the percentage of subjects with abnormal results considering the following age-dependent upper cut-off values of normal fecal calprotectin: 51 μg/g for subjects below 60 years of age, and 112 μg/g for subjects above 60 years (Joshi et al., 2009). Abnormal fecal calprotectin level was found in 43% of all PD patients and in none of the control subjects (*p* < 0.001). Dividing the PD patients into two age groups, abnormal fecal calprotectin level was observed in 50% of subjects below 60 years of age (6/12) and 39% of subjects above 60 years (9/23). The fecal zonulin level (ng/ml) was also higher in PD patients compared to the controls, but the *p*-value did not reach statistical significance: 161.85 (66.0–276.0) vs. 128.0 (66.8–264.2), *p* = 0.842. The mean disease duration, counted from the date of diagnosis, amounted to 9 years (from 1 to 20 years). No correlations between fecal calprotectin level and disease duration (*R* = –0.04, *p* = 0.832), MDS UPDRS score (*R* = 0.22, *p* = 0.218), and levodopa equivalent dose (*R* = –0.05, *p* = 0.774) were found.

The most frequent bowel symptoms reported by the PD patients included constipation (69% of subjects), feeling of incomplete evacuation (51%), bloating (51%), abdominal pain (20%), alternating bowel movement pattern (17%), and diarrhea (3%). Noteworthy, normal bowel habit was reported only by 11% of PD patients and all control subjects. The comparative analysis between two subgroups of PD patients: with normal and elevated fecal calprotectin level regarding the prevalence of the investigated bowel symptoms, PPI use, levodopa equivalent dose and MDS UPDRS III score did not reveal any significant differences (Table 2).

**TABLE 2:** The comparison between two subgroups of PD patients: with normal and elevated fecal calprotectin level.

	**PD patients with normal fecal calprotectin level *n* = 20**	**PD patients with elevated fecal calprotectin level *n* = 15**	***p*-value**
Constipation	60%	80%	0.207
Alternating bowel movement pattern	10%	27%	0.195
Incomplete evacuation	45%	60%	0.380
Abdominal pain	20%	20%	NA
Bloating	40%	67%	0.118
Proton pump inhibitor use	5%	20%	0.167
LED (mg) M [25Q–75Q]	625 [314–1015]	625 [500–937]	0.849
MDS UPDRS III M [25Q–75Q]	26.5 [21.0–47.5]	36.0 [29.0–44.0]	0.359

*M, median; MDS UPDRS, Movement Disorder Society Unified Parkinson’s Disease Rating Scale; LED, Levodopa Equivalent Dose.*

## Discussion

The results of the present study confirm that PD is characterized by the gut immune system activation. A large part of PD patients (43%), independently of the disease duration, exhibits signs of gut inflammation reflected by abnormal fecal calprotectin level. These data are consistent with the pioneer study by Devos et al. (2013) showing the association between PD and gut inflammation, in which the proinflammatory cytokine profile including TNF-α, IFN-γ, Il-6 and Il-1β strikingly corresponds to the changes observed in IBD. The results of the first pilot study in PD patients evaluating fecal calprotectin level, so far widely used as a marker of gut inflammation in the gastroenterology field, were reported by Mulak et al. (2016). Recently, those preliminary results have been confirmed and further expanded by reporting that not only calprotectin, but also α-1-antitrypsin and zonulin could be useful non-invasive fecal markers of intestinal inflammation and intestinal permeability in PD (Schwiertz et al., 2018). In our study, although we have seen some trend for higher fecal zonulin level in PD patients compared to the controls, we did not find statistically significant difference between the groups. Additionally, a very recent multivariate analysis of stool immune profiles revealed elevated levels of Il-1β, Il-1α, fecal C-reactive protein, and CXCL8 (Il-8) – a potent neutrophil chemoattractant and activator, that is in line with previous findings (Houser et al., 2018).

Calprotectin, a dimer of calcium binding proteins S100A8 and S100A9, constitutes a main component of the neutrophil protein content (up to 60%) and exerts bacteriostatic and fungistatic effects (Walsham and Sherwood, 2016). Elevated fecal calprotectin level indicates the migration of neutrophils to the intestinal mucosa or even to the gut lumen in case of gut barrier disturbances. Due to the complex stability and resistance to enzymatic degradation, calprotectin can be easily measured in stool (Walsham and Sherwood, 2016). Interestingly, the S100A8 and S100A9 proteins due to their intrinsically amyloidogenic amino acid sequences can form amyloid oligomers and fibrils closely resembling amyloid polypeptides such as α-syn and amyloid β (Kowalski and Mulak, 2019). Possibly, the intestinal pool of calprotectin may contribute to amyloid fibril formation both in the enteric nervous system (ENS) and the central nervous system. This hypothesis is in line with earlier findings in patients with Alzheimer’s disease documenting the increased level of fecal calprotectin (Leblhuber et al., 2015) and S100A9 level in cerebrospinal fluid (Horvath et al., 2016). On the other hand, it has been recently discovered that α-syn is expressed during gastrointestinal inflammation and serves as a strong chemoattractant for neutrophils and monocytes as a part of normal immune response (Stolzenberg et al., 2017). However, in pathological conditions, associated e.g., with increased intestinal permeability, an excessive expression and/or accumulation of α-syn within the ENS may result in its intracellular deposits (Kelly et al., 2014).

One important aspect in interpreting alterations in fecal calprotectin level is its age-dependent reference ranges. In the elderly, the immune system hyperstimulation results in chronic state of inflammation called “inflammaging” (Frasca and Blomberg, 2016). It may be associated with low-grade state of gut mucosa inflammation evoked by age-related changes in the gut microbiota composition characterized by its decreased stability and diversity (Frasca and Blomberg, 2016; Quigley, 2017). Accordingly to the report by Joshi et al. (2009), we used the following upper limits for two age groups: 51 μg/g for subjects below 60 years of age, and 112 μg/g for subjects above 60 years. Applying these age-related cut-off values, the increased fecal calprotectin level was found in 43% of PD patients. In the study by Schwiertz et al. (2018), adopting the cut-off value of 50 μg/g for all subjects, abnormal fecal calprotectin level was observed in 47% of PD patients. In comparison, in our age-independent analysis of the results, 57% of PD patients had fecal calprotectin level exceeding 50 μg/g.

The majority of PD patients suffer from gastrointestinal symptoms among which, as in this report, constipation is the most prominent one affecting up to 70% of subjects (Felice et al., 2016). Recently, constipation as well as irritable bowel syndrome (IBS) characterized by abdominal pain associated with altered bowel habit have been both identified as the risk factors for PD (Lai et al., 2014; Stirpe et al., 2016). Two recent studies concordantly reported that IBS-like symptoms are present in 27.1% (Mishima et al., 2017) and 24.3% (Mertsalmi et al., 2017) of PD patients. In our study abdominal pain was present in 20% of patients compared to 30% reported by another study (Mertsalmi et al., 2017). Interestingly, the second most common and equally prevalent complaints in PD patients were bloating and feeling of incomplete evacuation (51% of subjects). These symptoms are very common in IBS as well. In particular, feeling of incomplete evacuation might be considered as a marker of visceral hypersensitivity, possibly associated with the gut-immune system activation and constituting a characteristic feature of the disorders of gut-brain interactions such as IBS (Drossman, 2016). Moreover, incomplete evacuation and excessive straining belong to the spectrum of defecatory dysfunction, which has been recently confirmed in PD using high resolution anorectal manometry (Su et al., 2016).

The presence of gut inflammation associated with alterations in the gut microbiota composition, gastrointestinal sensory-motor dysfunction, and increased intestinal permeability (“leaky gut”) may have important clinical implications in PD patients. In particular, calprotectin possessing intrinsic amyloidogenic properties may constitute a critical link between amyloid formation and neuroinflammatory cascades serving as a prospective diagnostic and therapeutic target (Kowalski and Mulak, 2019). Based on the current evidence, interventions aimed at modulating the immune system, either blocking microglia-derived inflammatory mediators or harnessing the peripheral immune cells may present effective strategies.

## Data Availability

The datasets generated for this study are available on request to the corresponding author.

## Ethics Statement

The studies involving human participants were reviewed and approved by Ethics Committee at Wrocław Medical University, Wrocław, Poland (KB-491/2017). The patients or participants provided their written informed consent to participate in this study.

## Author Contributions

AM designed the study and wrote the manuscript. MK and SB wrote and revised the manuscript. All authors collected and interpreted the data and approved the final version of the manuscript.

## Conflict of Interest Statement

The authors declare that the research was conducted in the absence of any commercial or financial relationships that could be construed as a potential conflict of interest.
